# Emotional Context Modulates Neural Prediction‐Error Encoding

**DOI:** 10.1111/psyp.70420

**Published:** 2026-10-05

**Authors:** Darin R. Brown

**Affiliations:** ^1^ Department of Psychology Pitzer College USA

**Keywords:** EEG, emotion, prediction error, reinforcement learning

## Abstract

Emotional experiences are closely intertwined with reinforcement learning, yet the extent to which emotional context influences the neural encoding of learning signals remains unclear. The present study examined whether emotional context modulates trialwise neural prediction error (PE) encoding during reinforcement learning. Fifty‐six participants completed a drifting three‐armed bandit task while electroencephalography (EEG) was recorded. Choice options were consistently paired with positive, neutral, or negative emotional images that were presented during feedback processing. Trial‐by‐trial PEs were estimated using a computational reinforcement‐learning model and related to feedback‐locked EEG activity using hierarchical linear mixed‐effects modeling. Traditional condition‐averaged analyses revealed robust feedback‐related ERP responses to reward outcomes but minimal effects of emotional context on overt behavior or mean reward positivity (RewP) amplitude. In contrast, trialwise analyses demonstrated that emotional context significantly moderated the relationship between PE and feedback‐locked neural activity. The relationship between trialwise PEs and EEG amplitude was strongest in neutral contexts, whereas it was attenuated in positive emotional contexts during the early RewP interval and in negative emotional contexts during a later posterior stage of feedback processing. Consistent with this temporal pattern, scalp topographies revealed a corresponding shift from early frontocentral to later posterior activity over the course of feedback processing. Together, these findings suggest that emotional context does not substantially alter overt learning performance or condition‐averaged reward responses, but instead subtly shapes the neural encoding of PEs during reinforcement learning. More broadly, the results highlight the value of computationally informed trial‐level analyses for identifying affective influences on learning signals that may be obscured by traditional condition‐averaged approaches.

## Impact Statement

Traditional ERP analyses suggested that emotional context exerted little influence on reinforcement learning. However, computational trial‐level analyses revealed that emotional context dynamically modulated neural prediction‐error encoding across multiple stages of feedback processing. These findings demonstrate that affective influences on learning may emerge more clearly at the level of dynamic neural computation than in immediate behavioral adjustment or condition‐averaged neural responses, highlighting the value of computational approaches in psychophysiological research.

Reward acquisition is inherently an emotional experience. Obtaining desired outcomes elicits positive affect, whereas failing to obtain rewards or experiencing losses produces negative affective responses. These emotional reactions are not merely byproducts of reward processing but are tightly integrated with the neural systems that support learning and decision‐making (Phelps et al. 2014; Watanabe et al. 2013). Reinforcement learning theories posit that prediction errors (PE), discrepancies between expected and actual outcomes, serve as core teaching signals that guide behavior (Glimcher 2011; Sutton and Barto 1981). Converging evidence from cognitive neuroscience suggests that these learning signals are instantiated in neural responses such as the reward positivity (RewP), a feedback‐locked ERP component that differentiates rewarding from non‐rewarding outcomes (Brown et al. 2022; Cavanagh and Holroyd 2025; Holroyd and Coles 2002). Critically, because reward outcomes are inherently affective (Chiew and Braver 2011), an important open question is whether emotional context influences how these learning signals are encoded.

Reinforcement PE signals are thought to be represented in frontostriatal circuits (Niv et al. 2012) and can be indexed noninvasively using electroencephalography (EEG) (Cockburn and Holroyd 2018; Holroyd et al. 2008). The RewP, a frontocentrally distributed ERP component that emerges approximately 200 ms following outcome presentation (Brown et al. 2020; Proudfit 2015), is widely thought to reflect phasic reinforcement‐learning signals carried, at least in part, by the dopaminergic system (Cavanagh and Holroyd 2025; Proudfit 2015; Sambrook and Goslin 2015). Consistent with this account, pharmacological enhancement of dopaminergic signaling has been shown to increase RewP amplitude in humans (Cavanagh et al. 2022). Larger RewP amplitudes are typically observed for rewarding relative to non‐rewarding outcomes (Proudfit 2015), and compelling evidence suggests that trial‐by‐trial fluctuations in RewP amplitude track PE magnitude (Hoy et al. 2021; Lohse et al. 2020). Although the RewP is commonly associated with rapid reward evaluation and PE processing, later feedback‐related ERP components such as the feedback‐P3/P3b are thought to reflect subsequent evaluative, attentional, and context‐updating processes (Polich 2017; Sambrook and Goslin 2015). These feedback‐related components overlap temporally and may jointly contribute to how learning signals evolve over time during outcome processing. Neural indices of reinforcement learning may therefore provide a more sensitive measure of learning processes than overt behavior alone, as neural computations can vary even in the absence of large behavioral differences (Pagan et al. 2025; Rubin et al. 2019). This distinction is particularly relevant because PE signals contribute to the updating of value representations that can subsequently guide action selection (Nasser et al. 2017), but changes in these evaluative signals need not necessarily produce immediate changes in overt choice (Chase et al. 2010). Thus, examining neural PE encoding alongside behavioral learning provides an opportunity to determine whether contextual factors alter the evaluation and updating of outcomes even when their downstream influence on action selection is limited.

Given that reward outcomes are inherently affective, a critical question is whether emotional context influences how PE signals are encoded. Prior research has demonstrated that emotional stimuli can modulate attention (Yiend 2010), perception (Shalev and Sheva 2025), cognitive control (Chiew and Braver 2011; Xue et al. 2013; Zhang et al. 2023), and reward processing (Brown and Cavanagh 2018; Sander and Nummenmaa 2021) often biasing processing toward emotionally salient information. However, evidence for the role of emotion in reinforcement learning remains mixed (Pike and Robinson 2022). Some studies suggest that affective states alter reward sensitivity or learning rates (Schmidt et al. 2014), whereas others report minimal or inconsistent effects on behavior (Byrne et al. 2023; Harmon‐jones et al. 2012; Pulcu et al. 2024). These inconsistencies raise the possibility that emotional influences on reinforcement learning may emerge more strongly at the level of neural computation than in overt behavioral performance (Emanuel and Eldar 2023; Harlé et al. 2013; Watanabe et al. 2013). Recent work further suggests that reward‐related and affective/evaluative learning signals may unfold along partially distinct temporal neural trajectories during feedback processing (Heffner et al. 2025), with earlier frontocentral activity tracking reward PEs and later posterior activity reflecting affective or evaluative updating processes. These findings raise the possibility that emotional context may influence reinforcement learning not simply through broad changes in reward responsiveness, but through more subtle alterations in the temporal dynamics of neural PE encoding. Notably, much of the existing literature has focused on condition‐averaged behavioral or neural responses, which may obscure more subtle effects of emotion on the trial‐by‐trial computational mechanisms underlying learning (Teoh et al. 2026).

One possibility is that emotional context does not broadly amplify or suppress reward‐related neural activity, but instead alters how learning signals are represented over time (Katahira et al. 2015). In this perspective, emotion may modulate the temporal and spatial dynamics through which neural PE signals are encoded and evaluated over time, rather than the overall magnitude of reward‐related activity (Puccetti et al. 2022; Vogel et al. 2015). Such effects may be difficult to detect using traditional condition‐averaged analyses (Sajda et al. 2009), but may emerge when examining trial‐level relationships between PE and neural activity (Heffner et al. 2025).

The present study examined whether emotional context moderates the trial‐by‐trial relationship between PE and feedback‐locked neural activity during reinforcement learning. Participants completed a reinforcement‐learning task in which outcomes were embedded within positive, neutral, or negative emotional contexts while EEG was recorded. Trial‐by‐trial PE values were estimated using a computational learning model and related to feedback‐locked neural responses using hierarchical linear mixed‐effects modeling. In addition, computational reinforcement‐learning models were used to examine whether emotional context influenced overt learning behavior. Based on previous findings linking emotion and reward processing, emotional context was expected to influence conventional reward‐related ERP measures. However, it was hypothesized that emotional modulation would be more sensitively captured by trial‐level analyses. Specifically, it was predicted that emotional context would moderate the relationship between computationally derived trial‐by‐trial PE values and feedback‐locked neural activity. More broadly, the present study tested whether computationally informed trial‐level analyses provide a more sensitive framework for identifying emotional influences on reinforcement‐learning signals than traditional condition‐averaged approaches.

## 1 Method

### 1.1 Participants

Fifty‐six participants (38 female; M_age_ = 19.08 years, SD = 1.05) were recruited from the Pitzer College subject pool. Sample size was determined using an a priori power analysis conducted in G*Power version 3.1.9.7 (Faul et al. 2007). Assuming a medium effect size (partial η_p_^2^ = 0.05), *α* = 0.05, and power = 0.95, the analysis indicated a minimum required sample size of *N* = 51. Accordingly, 56 participants were recruited to ensure adequate statistical power.

Participants self‐reported their racial/ethnic identity as follows: 14% Latino/Hispanic, 36% Asian, and 50% non‐Hispanic White. Exclusion criteria included a history of head injury involving loss of consciousness greater than five minutes, a diagnosis of epilepsy, or current use of psychiatric or neurological medications. Participants received course credit for their participation. All procedures were approved by the Pitzer College Institutional Review Board.

### 1.2 Procedure

#### 1.2.1 Training Phase

Participants completed a probabilistic reinforcement learning task while EEG was recorded (Figure 1a). The task consisted of 300 trials divided into three blocks of 100 trials each, with self‐paced breaks provided between blocks.

**FIGURE 1:**
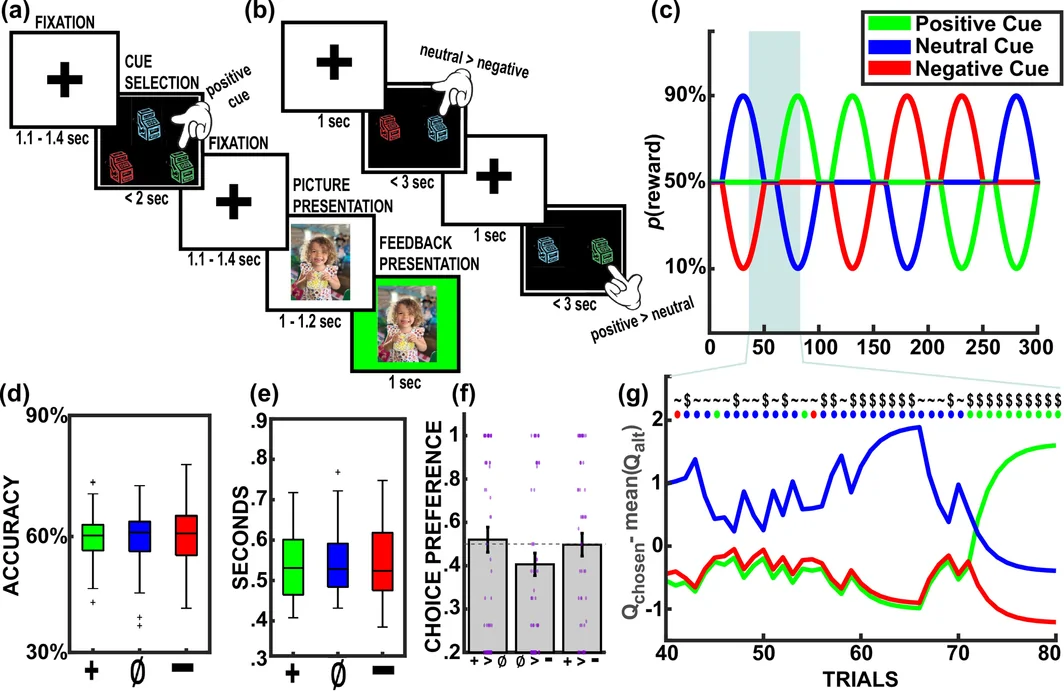
Three‐armed bandit task design, reward contingencies, and behavioral performance. (a) Trial structure during the training phase. Participants selected one of three slot machines, each consistently paired with a positive, neutral, or negative emotional cue. Following a jittered fixation period, an emotional image was presented, followed by reward feedback indicating a win or loss. Emotional images were task‐irrelevant and participants were explicitly informed that they were unrelated to reward outcomes. (b) Example testing‐phase trial. Participants selected between pairs of previously learned slot machines without receiving feedback. Testing trials assessed stable value preferences acquired during training. (c) Example reward‐probability schedules used during the training phase. Reward probabilities drifted throughout learning, such that one machine became highly rewarding (~90%), one became weakly rewarding (~10%), and one remained near chance (~50%). Machine–emotion pairings were counterbalanced across participants. (d) Accuracy during the training phase as a function of emotional condition. (e) Reaction times during the training phase as a function of emotional condition. No significant effects of emotional condition were observed for either measure. (f) Testing‐phase performance for each pairwise machine comparison. Points represent individual participants and bars represent means ± standard error of the mean. (g) Example reinforcement‐learning model output illustrating trial‐by‐trial value updating (Q‐values) for each slot machine across training. Colored dots indicate obtained outcomes and symbols above the plot denote trial feedback.

On each trial, participants were first presented with a fixation cross for a jittered interval of 1000–1400 ms. This was followed by the presentation of three slot machines arranged in a triangular configuration. Participants were instructed to select one of the three machines using the arrow keys on the keyboard (up, left, or right) within a limited response window (< 2000 ms). Failure to respond within this time resulted in a penalty of −3 points.

Immediately following each choice, a second fixation cross was presented (1000–1400 ms, jittered), followed by the presentation of an emotional image (1000–1200 ms, jittered). Images were selected from the International Affective Picture System (IAPS; Lang et al. 2005). Positive and negative images were chosen to differ in normative valence ratings while being matched on normative arousal, allowing emotional valence to vary independently of arousal. Neutral images were selected to have valence ratings near the midpoint of the scale and low arousal ratings. A one‐way ANOVA confirmed significant differences in valence across conditions, *F*(2, 297) = 5424.00, *p* < 0.001, with all pairwise comparisons differing significantly (Tukey‐adjusted *p*s < 0.001). A corresponding analysis of arousal ratings also revealed a significant effect of condition, *F*(2, 297) = 616.60, *p* < 0.001. Follow‐up comparisons indicated that positive and negative images did not differ in arousal (*p* = 0.796), whereas both emotional conditions were significantly more arousing than neutral images (Tukey‐adjusted *p*s < 0.001). Emotional images were categorized as positive, neutral, or negative, and each slot machine was consistently paired with one emotional category throughout the task. Critically, participants were explicitly informed that the emotional images were unrelated to reward outcomes and provided no information regarding reinforcement contingencies.

Feedback was presented immediately following image presentation. The background color of the screen changed to green to indicate a reward (+1 point) or red to indicate a loss (−1 point), while the image remained on the screen for 1000 ms. Feedback timing was fixed across trials.

The reinforcement structure followed a drifting three‐armed bandit design (Figure 1c). All slot machines began with equal reward probabilities (50%) for roughly 10 trials. Over the course of the task, reward probabilities gradually drifted such that the relative likelihood of reward associated with each machine changed continuously across trials. At any given period, one machine tended to increase in reward probability, one tended to decrease, and one remained relatively stable before contingencies gradually shifted again. After approximately 40 trials, reward probabilities gradually returned toward baseline and the process repeated. Participants were informed that reward contingencies could change over time and were encouraged to use feedback to guide their choices. Prior to the main task, participants completed a practice session in which positive, neutral, and negative emoji faces were paired with feedback to familiarize them with the task structure. Participants were allowed to repeat the practice if needed. The entire task took an average of 30.20 min (SD = 1.44).

#### 1.2.2 Testing Phase

Following the reinforcement‐learning phase, participants completed a testing phase designed to assess whether stable value preferences had developed for the three emotional slot machines (Figure 1b). On each trial, a fixation cross was presented for 1000 ms, followed by the presentation of two slot machines positioned side‐by‐side on the left and right sides of the screen. Participants were instructed to indicate, via button press, which of the two machines they believed was more rewarding overall based on their prior learning experience. Responses were required within 3000 ms. No feedback was provided during the testing phase, allowing assessment of learned value preferences in the absence of additional reinforcement. Each possible machine pairing (Positive vs. Neutral, Positive vs. Negative, Neutral vs. Negative) was presented eight times, with each machine appearing equally often on the left and right side of the screen (four presentations per side), resulting in a total of 24 trials. Pair presentation order was counterbalanced across participants.

### 1.3 Reinforcement Learning Model

Trial‐by‐trial PE values were estimated using a standard Q‐learning model. For each trial, expected values (Q) were updated according to:

$$Q\left(t+1\right)=Q\left(t\right)+\alpha \cdot \left(r\left(t\right)-Q\left(t\right)\right)$$

where r(t) represents the outcome on trial t (+1 for reward, −1 for loss), and (r(t) − Q(t)) corresponds to the PE. Separate value estimates were maintained for each emotional stimulus category (positive, neutral, negative). Model parameters were estimated separately for each participant by minimizing the negative log‐likelihood of observed choices using nonlinear optimization procedures implemented in MATLAB. The resulting participant‐specific parameters were then used to generate trial‐level PE estimates for each feedback event.

Following model fitting, PE values were generated for each trial using participant‐specific parameters. PEs were z‐scored within participants prior to statistical analysis to account for individual differences in scale. PE values were aligned with feedback‐locked EEG epochs. Feedback‐related EEG activity was primarily examined within the canonical RewP time window (200–300 ms) at electrode Cz. Additional exploratory analyses examined later posterior feedback‐related activity at electrode Pz and across scalp‐wide topographic distributions. Trials with missing EEG or behavioral data were excluded from analysis. Additionally, individual trials containing extreme EEG amplitudes within the RewP time window (mean amplitude exceeding ±40 μV at Cz during the 200–300 ms post‐feedback interval) were removed prior to statistical modeling.

To examine whether emotional context influenced overt reinforcement learning behavior, model comparisons were conducted between a standard Q‐learning model containing a single learning‐rate parameter and an emotion‐specific model containing separate learning‐rate parameters for positive, neutral, and negative conditions. Model fit was evaluated using Akaike Information Criterion (AIC), Bayesian Information Criterion (BIC), and likelihood‐ratio tests.

### 1.4 Data Acquisition and Preprocessing

EEG data were recorded using 32 Ag/AgCl electrodes mounted in a stretch‐lycra cap (Brain Products GmbH, Munich, Germany). Signals were sampled at 500 Hz with online bandpass filtering from 0.01 to 100 Hz. Cz served as the reference electrode and Fpz as the ground. Vertical electrooculogram (VEOG) activity was recorded using electrodes placed above and below the left eye to monitor blinks.

EEG preprocessing was conducted in MATLAB (MathWorks Inc 2022) using EEGLAB functions (Delorme and Makeig 2004). Continuous data were epoched from −1000 to 3000 ms relative to feedback onset (i.e., background color change). Data were re‐referenced to the average reference. A bandpass filter of 0.01–20 Hz was applied for ERP analyses. Artifact detection was conducted using the FASTER algorithm (Nolan et al. 2010), which identifies statistical outliers across channels and epochs. Using the runica algorithm (Makeig et al. 1996), independent component analysis was used to identify and remove components associated with eye blinks and other artifacts. ERP waveforms were computed by averaging across feedback trials. The RewP was quantified as the mean amplitude within a 200–300 ms time window following feedback onset at electrode site Cz. This interval was selected based on prior work indicating that the RewP emerges approximately 200 ms following outcome presentation and captures early reward‐related processing prior to the emergence of later feedback‐related positivities (Brown et al. 2020; Proudfit 2015). Additional exploratory analyses examined feedback‐related activity within a later posterior temporal window (300–500 ms) at electrode site Pz and across scalp‐wide topographic distributions of PE‐related EEG activity.

### 1.5 Statistical Analysis

Group‐level behavioral and ERP analyses were conducted using repeated‐measures analyses of variance (ANOVAs). Accuracy and reaction time were each analyzed using one‐way repeated‐measures ANOVAs with Emotion (positive, neutral, negative) as a within‐subject factor. Feedback‐related ERP amplitudes (RewP) were analyzed using a 3 (Emotion: positive, neutral, negative) × 2 (Outcome: win, loss) repeated‐measures ANOVA. When appropriate, Greenhouse–Geisser corrections were applied to adjust for violations of sphericity. Effect sizes for ANOVA results are reported as partial eta squared (ηp^2^). Hierarchical linear mixed‐effects models are summarized using fixed‐effect regression coefficients (*β*), test statistics (*F* or *t*, as appropriate), and associated *p* values.

Trial‐level relationships between PE and feedback‐locked EEG activity were examined using linear mixed‐effects models implemented in MATLAB (*fitlme*). EEG amplitude within the RewP time window (200–300 ms at Cz) was modeled as a function of z‐scored PE (PE_z_), emotional condition, and their interaction. The analysis included both win and loss trials; consequently, the dependent measure was single‐trial EEG amplitude rather than a win‐minus‐loss difference‐wave score. PE was entered as a continuous trial‐level predictor across both outcome types. Mixed‐effects models initially included random intercepts and random slopes for PE to account for between‐subject variability in baseline neural responses and PE sensitivity. Alternative random‐effects structures were evaluated when necessary to ensure model convergence and model stability. The primary model was specified as:

$$\text{RewP}∼{\mathrm{PE}}_{z}\times \text{Emotion}+\left(1+{\mathrm{PE}}_{z}|\text{Subject}\right)$$

To determine whether the relationship between PE and neural activity differed across win and loss trials, an additional mixed‐effects model included outcome type (win vs. loss) and all associated interactions. Follow‐up models were conducted separately for win and loss trials to characterize outcome‐specific PE × Emotion relationships:

$$\text{RewP}∼{\mathrm{PE}}_{z}\times \text{Emotion}\times \text{Outcome}+\left(1+{\mathrm{PE}}_{z}|\text{Subject}\right)$$

To assess whether emotional modulation of trialwise PE encoding extended beyond the canonical RewP interval, an analogous linear mixed‐effects model was computed within a later posterior temporal window (300–500 ms) at electrode site Pz. EEG amplitude within this interval was modeled as a function of PE_Z_, emotional condition, and their interaction. As with the primary RewP analyses, this model included both win and loss trials. To examine whether the later PE × Emotion relationship was moderated by outcome type, an additional model including the PE × Emotion × Outcome interaction was also evaluated. Follow‐up models were conducted separately for win and loss trials to characterize outcome‐specific PE × Emotion relationships. The primary model was specified as:

$$\mathrm{Pz}\;\left(300-500\right)∼{\mathrm{PE}}_{z}\times \text{Emotion}+\left(1+{\mathrm{PE}}_{z}|\text{Subject}\right)$$

To further characterize emotion‐specific relationships between PE and neural activity, additional visualization analyses were conducted at the subject, temporal, and spatial levels. Subject‐level linear regressions relating trialwise PE to feedback‐locked EEG amplitude were computed separately for each emotional condition, and resulting regression coefficients were used to visualize PE–EEG relationships across participants. To examine the temporal dynamics of PE encoding, single‐trial EEG amplitude was regressed onto trialwise PE values at each feedback‐locked time point separately for each emotional condition. As a follow‐up analysis, these time‐resolved regressions were additionally performed separately for win and loss trials to characterize potential outcome‐specific effects. Resulting beta‐weight waveforms were averaged across participants to visualize the time course of neural PE encoding across emotional contexts. Exploratory one‐sample *t*‐tests against zero were conducted at each time point within each emotional condition to identify periods during which PE beta weights significantly differed from zero. Finally, exploratory analyses examined scalp‐wide topographic distributions of PE beta weights within predefined RewP (200–300 ms) and later posterior (300–500 ms) temporal windows.

To examine whether trial‐to‐trial computational and neural signals were associated with subsequent behavioral adjustments, additional mixed‐effects analyses were conducted. First, log‐transformed reaction time on trial n was modeled as a function of the previous trial's PE (trial *n* − 1), the emotional condition of the current trial, and their interaction, with a random intercept for participant. This analysis paralleled the primary trial‐level neural models while accounting for the temporal ordering of PEs and subsequent behavioral responses. Second, log‐transformed reaction time on trial n was modeled as a function of later posterior EEG amplitude (300–500 ms at Pz) from trial *n* − 1, the previous trial's emotional condition, and their interaction. Finally, a generalized linear mixed‐effects model examined whether participants repeated or switched their previous choice on trial n as a function of the previous trial's PE, emotional condition, and their interaction.

## 2 Results

### 2.1 Reinforcement Learning Training Results

Descriptive statistics for experimental measures are reported in Table 1, and behavioral performance is illustrated in Figure 1d,e. A repeated‐measures ANOVA revealed no significant effect of emotional condition on accuracy, *F*(1.97, 108.37) = 0.152, *p* = 0.859, η_p_^2^ = 0.003, indicating comparable performance across emotional conditions. Similarly, reaction time did not significantly differ as a function of emotion, *F*(1.82, 99.95) = 1.214, *p* = 0.299, η_p_^2^ = 0.022, suggesting that emotional context did not influence basic task performance.

**TABLE 1:** Means and Standard Deviations for Experimental Measures.

Condition	Accuracy *(%)*	RT (seconds)	RewP WIN (μV)	RewP LOSS (μV)	PE *β* (μV per z‐scored PE)
Positive	59.9 (5.8)	0.54 (0.08)	3.99 (2.32)	3.26 (2.73)	−0.14
Neutral	60.0 (6.8)	0.54 (0.08)	3.82 (2.46)	3.33 (2.86)	0.28
Negative	60.3 (8.0)	0.55 (0.09)	3.63 (2.33)	3.07 (2.63)	0.14

*Note:* Values are reported as M (SD). Accuracy values are percentages. RewP amplitudes were quantified at Cz within the 200–300 ms window following feedback onset. PE *β* values represent condition‐specific slopes relating trialwise PEs to feedback‐locked EEG amplitude estimated from the mixed‐effects model.

To further examine whether emotional context influenced reinforcement learning behavior, computational model comparisons were conducted between a standard Q‐learning model containing a single learning‐rate parameter and an emotion‐specific model containing separate learning‐rate parameters for positive, neutral, and negative conditions. The emotion‐specific model showed modest improvement in fit according to AIC for 30 of 56 participants; however, the simpler model was preferred according to the more conservative BIC for the majority of participants. Likelihood‐ratio tests indicated significantly improved fit for the emotion‐specific model in 20 participants. Collectively, these findings suggest that emotional context exerted only modest and heterogeneous influences on overt learning behavior.

### 2.2 Reinforcement Learning Testing Results

Testing‐phase performance is illustrated in Figure 1e. To assess whether participants developed stable value preferences during learning, choice behavior during the testing phase was examined. For each machine pair, the proportion of choices favoring the objectively more rewarding machine was calculated. Pairwise comparisons revealed no significant differences in the proportion of higher‐value choices between the Positive–Neutral (*p* = 0.731) and Positive–Negative (*p* = 0.957) pairings. The Neutral–Negative comparison approached significance (*p* = 0.077) but did not reach conventional statistical thresholds. Furthermore, overall testing performance did not significantly exceed chance levels (*p* = 0.500). A complementary nonparametric sign‐rank test produced a similar result (*p* = 0.504). Together, these findings suggest that emotional context exerted minimal influence on stable overt value preferences during the testing phase.

### 2.3 Feedback‐Related ERP Effects

Condition‐averaged feedback‐locked ERP waveforms are displayed in Figure 2. Mean RewP amplitudes were analyzed using a 3 (Emotion: positive, neutral, negative) × 2 (Outcome: win, loss) repeated‐measures ANOVA. A significant main effect of outcome was observed, *F*(1, 55) = 12.501, *p* < 0.001, η_p_^2^ = 0.185, such that win trials elicited larger RewP amplitudes than loss trials. There was no main effect of emotion, *F*(1.79, 98.46) = 1.644, *p* = 0.198, η_p_^2^ = 0.029, and no Emotion × Outcome interaction, *F*(1.86, 102.42) = 0.375, *p* = 0.688, η_p_^2^ = 0.007. These results indicate that feedback valence robustly modulated neural responses, whereas emotional context did not significantly influence mean RewP amplitudes in the condition‐averaged ERP analyses.

**FIGURE 2:**
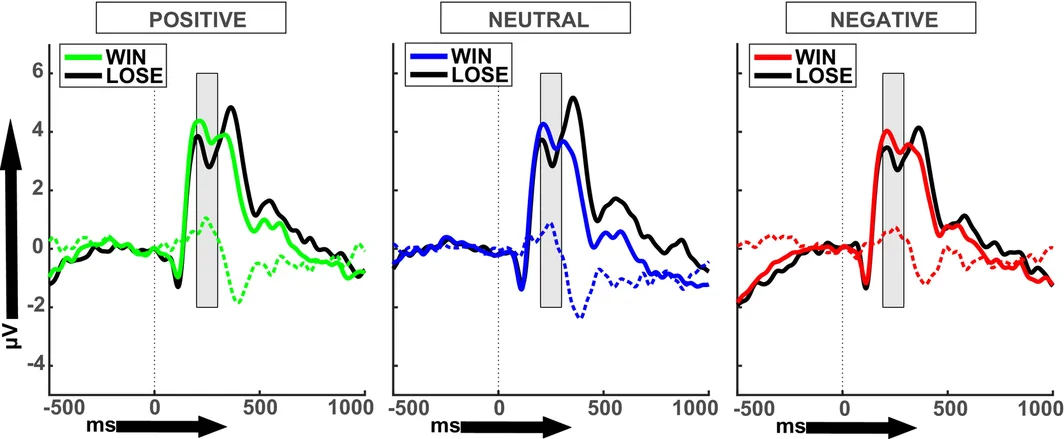
Condition‐averaged feedback‐locked ERPs across emotional contexts. Grand‐average event‐related potentials (ERPs) recorded at electrode Cz are shown separately for positive (left), neutral (middle), and negative (right) emotional conditions. Solid colored lines represent win trials, black lines represent loss trials, and dashed colored lines represent win‐minus‐loss difference waves (RewP) for each emotional condition. The gray shaded region denotes the 200–300 ms RewP time window used for mean‐amplitude quantification. Across emotional conditions, win outcomes elicited larger positive‐going neural responses than loss outcomes, consistent with canonical RewP effects. Visual inspection suggests broadly similar feedback‐locked ERP responses across emotional contexts, consistent with the absence of significant emotion‐related effects in the condition‐averaged analyses.

### 2.4 Trial‐Wise Prediction Error Effects

#### 2.4.1 Early Prediction Error Encoding (200–300 Ms)

To examine whether feedback‐locked EEG activity tracked computational learning signals, trial‐level EEG data were analyzed across all feedback trials using hierarchical linear mixed‐effects models implemented in MATLAB. Feedback‐locked EEG amplitude was predicted from PE_Z_, emotional condition, and their interaction while accounting for repeated observations nested within participants. This analysis revealed a significant main effect of PE, *F*(1, 16,095) = 5.62, *p* = 0.018, indicating that larger PEs were associated with greater feedback‐locked EEG amplitudes. Denominator degrees of freedom reflect the total number of trial‐level observations included in the mixed‐effects model.

A significant main effect of emotional condition was also observed, *F*(2, 16,095) = 5.64, *p* = 0.004. Critically, emotional context significantly moderated the relationship between PE and feedback‐locked EEG activity, as evidenced by a significant PE × Emotion interaction, *F*(2, 16,095) = 3.57, *p* = 0.028. Follow‐up tests of the PE slopes indicated that PE significantly predicted RewP amplitude in the neutral condition (*β* = 0.28, *p* = 0.018), but not in the positive (*β* = −0.14, *p* = 0.198) or negative (*β* = 0.14, *p* = 0.228) conditions. Pairwise comparisons further demonstrated that the PE slope was significantly reduced in the positive condition relative to the neutral condition (*β* = −0.42, *p* = 0.009), whereas the negative condition did not differ significantly from the neutral condition (*β* = −0.14, *p* = 0.389) or the positive condition (*β* = 0.28, *p* = 0.078).

Additional visualization analyses were conducted to further characterize the temporal dynamics of these effects (Figure 3). Consistent with the mixed‐effects model, subject‐level regression slopes demonstrated that the neutral condition exhibited the strongest positive relationship between PE and feedback‐locked EEG activity, whereas this relationship was attenuated in positive emotional contexts and intermediate in negative contexts. Time‐resolved analyses demonstrated that PE‐related neural activity emerged prominently within the RewP interval (200–300 ms).

**FIGURE 3:**
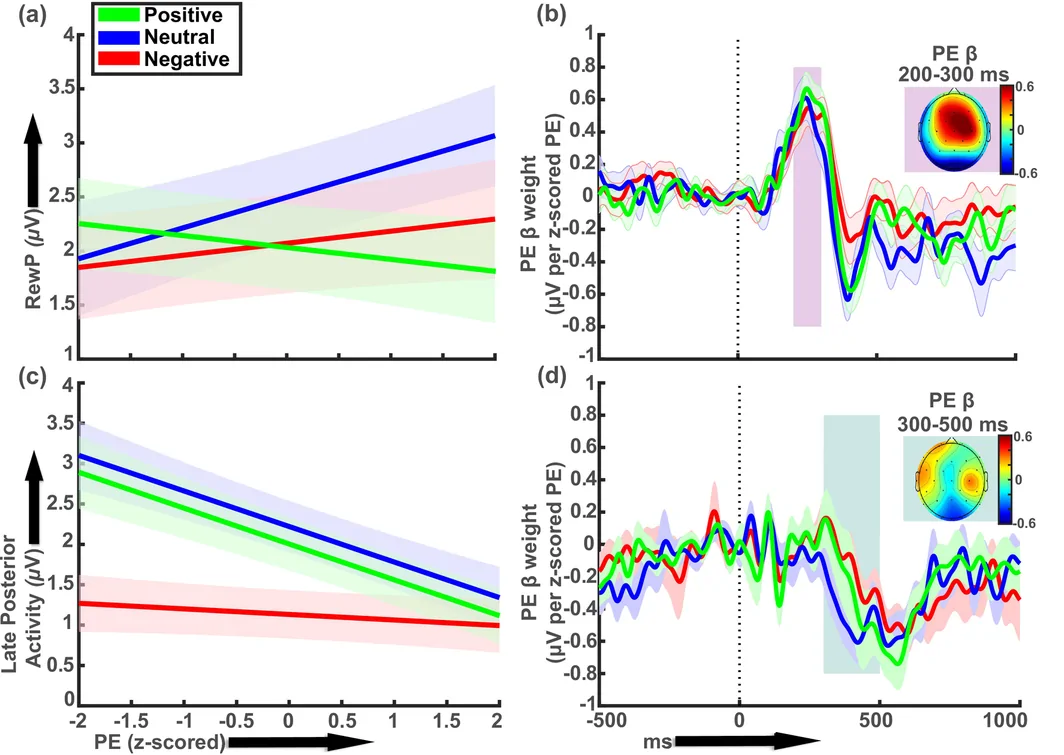
Emotional modulation of trialwise prediction‐error encoding across early and later stages of feedback processing. (a) Mean subject‐level relationship between trialwise prediction error (PE) and RewP amplitude (200–300 ms) as a function of emotional context. Shaded regions represent ±1 SEM across participants' predicted PE–RewP relationships. (b) Time‐resolved subject‐level regression coefficients (*β* weights relating z‐scored PE to EEG amplitude) across positive, neutral, and negative emotional contexts. Shaded regions represent ±1 SEM across participants' PE regression coefficients at each time point. The 200–300 ms RewP analysis window is highlighted in purple, and the inset depicts the scalp distribution of PE‐related activity during this interval. (c) Mean subject‐level relationship between trialwise PE and later posterior activity (300–500 ms) as a function of emotional context. Shaded regions represent ±1 SEM across participants' predicted PE–later posterior activity relationships. (d) Time‐resolved subject‐level PE regression coefficients across emotional contexts. Shaded regions represent ±1 SEM across participants' PE regression coefficients at each time point. The 300–500 ms later posterior analysis window is highlighted in blue, and the inset depicts the scalp distribution of PE‐related activity during this interval.

#### 2.4.2 Late Prediction Error Encoding (300–500 Ms)

To examine whether emotional modulation of PE signals was also evident within a later posterior temporal window (300–500 ms), additional mixed‐effects models were computed at electrode site Pz. PE significantly predicted later posterior EEG amplitude, *F*(1, 16,076) = 18.92, *p* < 0.001. A significant PE × Emotion interaction was also observed, *F*(2, 16,076) = 4.01, *p* = 0.018, indicating that emotional context continued to modulate trialwise PE encoding during later stages of feedback processing. Follow‐up analyses of the conditional PE slopes indicated that PE significantly predicted later posterior activity in the neutral (*β* = −0.43, *p* < 0.001) and positive (*β* = −0.38, *p* < 0.001) conditions, but not in the negative condition (*β* = −0.07, *p* = 0.436). Pairwise comparisons demonstrated that the negative condition exhibited a significantly weaker relationship between PE and later posterior activity than both the neutral (*β* = 0.35, *p* = 0.010) and positive (*β* = 0.31, *p* = 0.021) conditions, whereas the positive and neutral conditions did not differ from one another (*β* = 0.05, *p* = 0.731). Together, these findings indicate that emotional context continued to modulate trialwise PE encoding, with the negative emotional context exhibiting significantly weaker PE encoding than the positive and neutral contexts. Time‐resolved analyses further demonstrated that PE‐related neural activity extended beyond the RewP interval, while scalp topographies revealed a corresponding transition from an early frontocentral to a later posterior distribution (Figure 3).

#### 2.4.3 Outcome‐Specific Prediction‐Error Encoding

To evaluate whether emotional modulation of early PE encoding differed across win and loss trials, outcome type was added to the mixed‐effects model. The omnibus PE × Emotion × Outcome interaction did not reach statistical significance, F(2, 16,089) = 2.77, *p* = 0.063, indicating insufficient evidence that the influence of emotional context on early PE encoding differed reliably between wins and losses. Given the pattern of the omnibus interaction, exploratory analyses were conducted separately for win and loss trials. The PE × Emotion interaction was not significant for win trials, *F*(2, 9710) = 0.08, *p* = 0.920, but was significant for loss trials, *F*(2, 6379) = 5.16, *p* = 0.006, suggesting that emotional modulation of early PE encoding may have been more pronounced following losses. Figure 4 illustrates the time‐resolved PE–EEG relationships at Cz separately for win and loss trials. Although greater differentiation among emotional conditions was apparent following losses, the omnibus three‐way interaction was not statistically significant; accordingly, these outcome‐specific patterns are interpreted cautiously.

**FIGURE 4:**
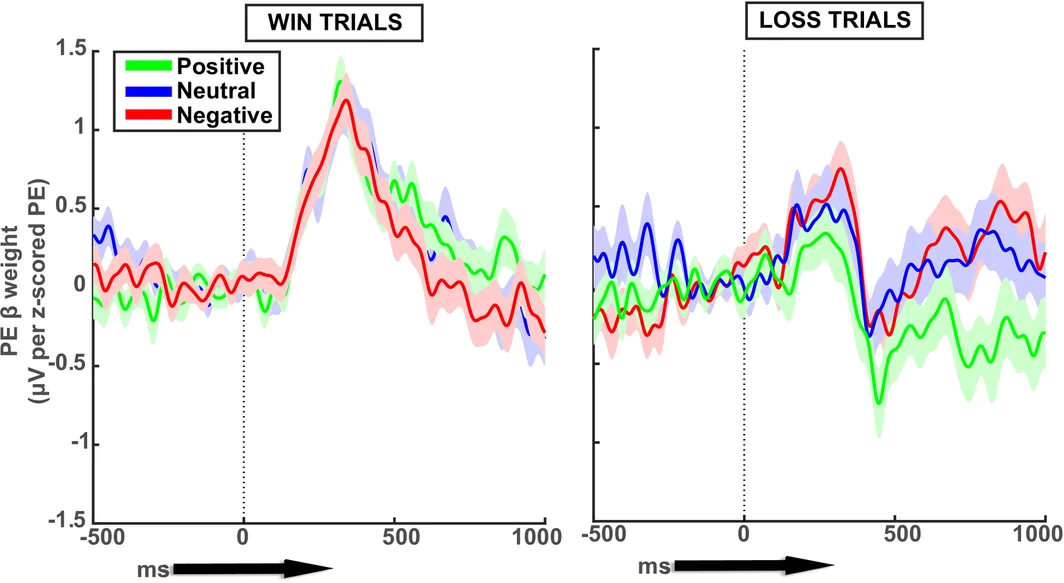
Outcome‐specific trialwise prediction‐error encoding at Cz across emotional contexts. Time‐resolved subject‐level regression coefficients (*β* weights relating z‐scored computational prediction error (PE) to EEG amplitude) at electrode Cz are shown separately for win trials (left panel) and loss trials (right panel). Green traces represent positive emotional contexts, blue traces represent neutral emotional contexts, and red traces represent negative emotional contexts. Shaded error bands represent ±1 SEM across participants' PE regression coefficients at each time point.

Emotional modulation of later posterior PE encoding at electrode Pz was subsequently examined across outcome types. The omnibus PE × Emotion × Outcome interaction was not significant, *F*(2, 16,070) = 0.33, *p* = 0.717, indicating insufficient evidence that the later PE × Emotion relationship differed reliably between win and loss trials. Consistent with this result, exploratory outcome‐stratified analyses yielded nonsignificant PE × Emotion interactions for both win trials, *F*(2, 9699) = 0.33, *p* = 0.716, and loss trials, *F*(2, 6371) = 0.23, *p* = 0.791. Thus, there was no evidence that emotional modulation of later posterior PE encoding differed reliably as a function of outcome type.

### 2.5 Trial‐To‐Trial Behavioral Analyses

A hierarchical linear mixed‐effects model revealed no main effect of previous PE, *F*(1, 15,866) = 0.46, *p* = 0.499, no main effect of emotion, *F*(2, 15,883) = 1.17, *p* = 0.309, and no Previous PE × Emotion interaction, *F*(2, 15,867) = 0.37, *p* = 0.692, on next‐trial log‐transformed reaction time. Thus, emotional context did not measurably influence next‐trial response speed despite modulating neural PE encoding.

Additional exploratory analyses examined whether later posterior neural activity predicted subsequent reaction time and whether previous PE predicted stay‐versus‐switch behavior. Later posterior neural activity did not predict subsequent reaction time, nor was this relationship moderated by emotional context. As expected, previous PE strongly predicted whether participants repeated or switched their previous choice, *F*(1, 15,921) = 230.76, *p* < 0.001, consistent with adaptive reinforcement learning; however, this relationship was not moderated by emotional context, *F*(2, 15,921) = 1.79, *p* = 0.167. Complete results from these exploratory analyses are provided in Table 2. Together, these analyses provided no evidence that the observed emotional modulation of neural PE encoding was accompanied by detectable immediate behavioral adjustments.

**TABLE 2:** Exploratory trial‐to‐trial analyses of subsequent behavioral adjustment.

Analysis	Predictor	*F*	df	*p*
Next‐trial RT	Previous later posterior activity	0.056	1, 25,869	0.813
	Previous emotion × previous later posterior activity	0.401	2, 15,870	0.670
Stay/Switch	Previous PE	230.76	1, 15,921	< 0.001
	Emotion × previous PE	1.788	2, 15,921	0.167

*Note:* F statistics and denominator degrees of freedom are reported from mixed‐effects models. “Previous later posterior activity” refers to mean EEG amplitude within the 300–500 ms window at electrode Pz on the preceding trial. Stay/Switch analyses were estimated using generalized linear mixed‐effects models.

## 3 Discussion

The present study examined whether emotional context modulates reinforcement‐learning signals at the level of feedback‐related neural processing. Although emotional cues exerted minimal influence on overt behavioral performance and did not significantly modulate condition‐averaged feedback‐locked ERP amplitudes, robust neural differentiation between reward and non‐reward outcomes was observed. Consistent with the primary hypothesis, however, emotional modulation was more sensitively detected using computationally informed trial‐level analyses. Specifically, emotional context significantly moderated the relationship between trial‐by‐trial PE and feedback‐locked EEG activity. PE robustly tracked neural activity in neutral contexts, whereas this relationship was attenuated in positive emotional contexts, with negative contexts showing an intermediate pattern. Interestingly, this pattern evolved across time, such that positive emotional contexts selectively attenuated early PE encoding within the RewP interval, whereas negative emotional contexts exhibited weaker PE encoding during the later posterior stage of feedback processing. Although exploratory outcome‐stratified analyses suggested that this relationship may have been more pronounced following losses than wins, the omnibus PE × Emotion × Outcome interaction was not statistically significant, indicating that this possibility should be interpreted cautiously until replicated. Together, these findings suggest that emotional context does not broadly alter reward‐related neural activity, but instead subtly shapes how learning signals are dynamically encoded and evaluated over time.

These findings align with emerging evidence suggesting that reinforcement learning signals unfold across multiple temporally and functionally distinct stages of feedback processing (Glazer et al. 2018; Heffner et al. 2025; Liu et al. 2025). Recent work has demonstrated that reward PEs and affective/evaluative learning signals may exhibit partially separable neural trajectories, with earlier frontocentral feedback activity tracking reward‐related PEs and later posterior activity reflecting affective or evaluative updating processes (Heffner et al. 2025). The present findings complement this perspective by demonstrating that even task‐irrelevant emotional context can subtly influence the neural encoding of reward PEs during reinforcement learning. Importantly, emotional modulation in the present study did not appear as a large condition‐averaged shift in ERP amplitude, but instead emerged through distributed changes in the trialwise relationship between PE and feedback‐locked neural activity over time.

Importantly, behavioral analyses revealed only weak and inconsistent effects of emotion on overt learning behavior. Emotional condition did not significantly influence choice accuracy or reaction time, and additional trial‐level analyses revealed no evidence that emotional context moderated the relationship between previous PE and subsequent reaction time. Computational model comparisons likewise provided only modest support for emotion‐specific learning rates. While the emotion‐specific reinforcement learning model improved fit for some participants, the simpler single‐learning‐rate model was generally preferred when accounting for model complexity. Together, these findings suggest that emotional context exerted limited influence on overt behavioral learning performance.

One factor that may have contributed to these modest behavioral and condition‐averaged ERP effects is that positive and negative images were intentionally matched on normative arousal ratings rather than selected to maximize emotional intensity. This conservative design minimized potential arousal confounds, allowing for a more specific examination of the contribution of emotional valence to reinforcement learning. Critically, however, robust effects emerged at the neural level when examining trial‐by‐trial PE encoding. This dissociation is theoretically important because it suggests that emotional influences on reinforcement learning may emerge more strongly at the level of neural computation than in observable behavior.

One possible interpretation of this neural–behavioral dissociation can be considered within an actor–critic framework of reinforcement learning (Houk et al. 1995). In such models, critic‐like processes evaluate outcomes and update value representations, whereas actor‐like processes use learned value information to guide action selection (O'doherty et al. 2004). The present finding that emotional context modulated feedback‐locked PE encoding without producing corresponding effects on overt learning behavior is consistent with the possibility that emotional context primarily influenced evaluative, critic‐like computations without sufficiently altering downstream action‐selection processes to produce detectable behavioral differences. This possibility is also consistent with the trial‐to‐trial behavioral analyses: although PE strongly predicted whether participants subsequently repeated or switched their previous choice, emotional context did not moderate this relationship. Importantly, however, the present Q‐learning model did not separately parameterize actor and critic processes, and these findings therefore cannot establish that emotional modulation occurred within critic‐like processes. Rather, the observed dissociation identifies this as an important possibility for future investigation.

In addition to characteristics of the stimulus set, individual differences in affective valuation may also have contributed to variability in emotion‐related reward processing. Consistent with this interpretation, previous work has demonstrated that individual differences in subjective preferences for affective images predicted individual differences in the RewP (Brown et al. 2022), suggesting that personal affective valuation may moderate emotion–reward interactions. Future studies incorporating individual ratings of emotional stimuli may help clarify whether subjective affective value moderates the influence of emotional context on trial‐by‐trial PE encoding.

Emotional context similarly exerted minimal influence on overt testing‐phase choice behavior. Participants did not reliably demonstrate stable preference differences between the emotional slot machines, and overall testing‐phase performance did not significantly exceed chance levels. Although initially surprising given the extensive literature demonstrating behavioral effects of emotion (Bartholomeyczik et al. 2022; Lench et al. 2013), this pattern may reflect the distinction between transient emotional influences on ongoing neural computations and stable overt behavioral preference formation (Heffner et al. 2025). In the current task, emotional stimuli were incidental to the reinforcement contingencies themselves, allowing participants to maintain stable reward‐maximization strategies despite subtle modulation of feedback‐related neural processing. This dissociation is theoretically important because it suggests that emotional context may shape how reinforcement‐learning signals are neurally processed without necessarily producing large or stable alterations in overt choice behavior (Engelmann et al. 2015; Kool et al. 2013). Together, these findings further support a dissociation between neural PE encoding and overt behavioral preference formation.

This neural–behavioral dissociation was also evident in the trial‐to‐trial analyses. Emotional context did not moderate the relationship between previous PE and subsequent reaction time, even when behavior was analyzed using the same trial‐level computational framework that revealed robust neural effects. Likewise, later posterior neural activity did not predict subsequent reaction time. Together with the absence of emotional modulation of PE‐related stay/switch behavior described above, these findings provide additional evidence that the observed neural effects were not accompanied by detectable trial‐to‐trial behavioral adjustments.

Additional analyses further suggested that emotional influences on PE processing were not restricted to the canonical RewP interval. PE‐related neural activity emerged prominently within the RewP window (200–300 ms), where scalp topographies exhibited the expected frontocentral distribution associated with feedback‐related reward processing (Tunison et al. 2019; Weber and Bellebaum 2024). During this early stage, PE robustly tracked neural activity in neutral contexts but was significantly attenuated in positive emotional contexts, suggesting that positive emotion selectively attenuated the neural representation of trialwise learning signals. Importantly, follow‐up mixed‐effects analyses demonstrated that PE continued to significantly predict neural activity within a later posterior temporal window (300–500 ms), where emotional modulation remained evident but exhibited a different pattern. Specifically, PE encoding remained robust in neutral and positive contexts but was significantly attenuated in negative emotional contexts. Scalp topographies were consistent with a shift from an early frontocentral distribution to a more posterior/parietal distribution over time. Together, these findings suggest that emotional influences on reinforcement‐learning signals evolve across multiple stages of feedback processing, with positive emotional context exerting its greatest influence during early reward evaluation and negative emotional context exhibiting a more pronounced influence during later stages associated with evaluation, context updating, and outcome integration.

These findings further highlight the value of computationally informed trial‐level analyses for studying reinforcement learning processes (Lohse et al. 2020; Stange et al. 2020). Traditional condition‐averaged ERP analyses revealed robust outcome effects but minimal evidence of emotional modulation, with feedback‐locked waveforms appearing highly similar across emotional conditions (Brown et al. 2022; Hoy et al. 2021; Jackson and Cavanagh 2023). In contrast, trialwise computational approaches revealed that emotional context altered how neural activity tracked deviations between expected and actual outcomes over time. This dissociation suggests that averaging neural responses across trials may obscure meaningful variability in the dynamic relationship between cognition, emotion, and learning. More broadly, the present findings support the idea that trial‐level computational analyses may provide a more sensitive framework for investigating how affective context shapes neural learning signals than condition‐averaged ERP approaches alone.

One interpretation of these findings is that emotional context alters the fidelity or organization of neural PE signaling, thereby subtly influencing how outcome information is represented during feedback evaluation (Jackson and Cavanagh 2023; Watanabe et al. 2013). During the early RewP interval, positive emotional contexts selectively attenuated the relationship between PE and neural activity, suggesting that positive affect may reduce the precision with which deviations between expected and actual outcomes are encoded. Exploratory outcome‐stratified analyses further suggested that this early emotional modulation may have been more apparent following losses than wins, raising the possibility that outcome valence may qualify the influence of emotional context on early PE encoding. However, the nonsignificant omnibus interaction precludes strong conclusions regarding outcome specificity. The broader pattern of attenuated PE encoding in positive emotional contexts aligns with theoretical accounts proposing that positive affect promotes broader or more heuristic‐based processing styles, potentially at the expense of fine‐grained monitoring of environmental discrepancies (Isbell et al. 2013). Under such conditions, neural systems supporting reinforcement learning may prioritize maintenance of a positive affective state over precise encoding of outcome deviations (Vollberg and Sander 2024). Importantly, the present findings suggest that emotional context does not simply amplify or suppress reward processing globally, but instead changes how computationally relevant information is represented during feedback processing.

The influence of emotional context was not uniform across the course of feedback processing. Although positive emotional contexts selectively attenuated PE encoding during the early RewP interval, later posterior activity exhibited a different pattern, with PE encoding remaining robust in positive and neutral contexts but significantly attenuated in negative emotional contexts. This temporal dissociation suggests that positive and negative emotional states may exert distinct influences on different stages of reinforcement learning. Taken together, these findings raise the possibility that positive and negative affect influence reinforcement learning through partially distinct mechanisms. Positive affect may bias the initial computation of PEs toward broader, more heuristic processing, whereas negative affect may exert greater influence during later stages of outcome evaluation, context updating, or affective integration (Lefebvre et al. 2017; Roese and Olson 2007). Alternatively, the later posterior activity may reflect cognitive processes that are inherently more sensitive to negatively valenced information than the initial frontocentral PE signal.

An alternative computational framework offers a different perspective on the pattern of findings observed across both temporal stages of feedback processing. Rather than directly altering the fidelity of neural PE encoding, the positive emotional images themselves may have functioned as rewarding events, transiently increasing the affective value of the feedback context. Under this account, monetary wins occurring in positive emotional contexts would be evaluated against an already elevated reward baseline, resulting in smaller apparent neural PE signals during the early RewP interval. From a reinforcement‐learning perspective, emotional stimuli may therefore alter the estimated value of the current state, thereby indirectly influencing subsequent PE signals rather than directly changing the fidelity of PE encoding itself (Katahira et al. 2015). Although this framework most naturally accounts for the early attenuation of PE encoding, it also raises the possibility that the later posterior effects reflect downstream consequences of an initially altered value representation, rather than an independent mechanism. Specifically, if emotional stimuli first modify state‐value representations during early feedback evaluation, later posterior activity may reflect downstream processes involved in integrating those altered value representations with broader contextual or affective information. Although the present design cannot distinguish between these mechanisms, both frameworks converge on the broader conclusion that emotional context shapes the neural computations supporting reinforcement learning (Gruber and McDonald 2012). These competing accounts generate testable predictions for future work. If emotional context alters state‐value representations, emotional information incorporated into the predictive context before outcome delivery should systematically shift estimated value and, consequently, the magnitude of subsequent PEs. In contrast, if emotion primarily alters the fidelity of neural PE encoding, emotional context should moderate the relationship between computationally estimated PE and neural activity even when emotional information does not alter the value representation from which the PE is derived. Future studies manipulating the timing of emotional information relative to outcome delivery, while modeling state‐value estimates and neural PE signals separately, could therefore help distinguish between these possibilities. Source‐localization and time‐frequency approaches could further determine whether the early and later effects reflect distinct neural processes or downstream stages of a common affectively modulated learning signal.

The present findings contribute to a growing literature emphasizing that emotion and cognition interact at the level of computational mechanisms rather than through broad additive effects on behavior or neural activity alone (John et al. 2013). By demonstrating that emotional context selectively alters the relationship between PE and neural response, the current study suggests that affective information can shape how learning signals are encoded even when overt performance remains relatively stable. More broadly, the findings highlight the value of integrating computational modeling with electrophysiological methods to investigate how emotional context shapes learning and decision‐making processes.

Several limitations of the present study should be noted. First, emotional stimuli were task‐irrelevant and were presented only after decisions had already been made, which may have reduced their influence on overt learning behavior. Emotional context may exert stronger effects during earlier stages of reinforcement learning, including anticipatory value representation, motivational engagement, and action selection prior to outcome presentation (Dayan and Balleine 2002). In the current task, emotional influences were therefore largely restricted to feedback evaluation itself. The actor–critic interpretation of the present neural–behavioral dissociation also generates a testable prediction for future work. If incidental emotional context primarily modulates critic‐like evaluative processes, emotional stimuli presented only during feedback should preferentially alter neural PE signals and value‐updating processes while exerting comparatively weak effects on subsequent action selection. In contrast, making emotional information motivationally relevant to the available actions, for example, by associating emotional stimuli with specific choice options or reward contingencies prior to choice, should increase the likelihood that affectively altered value representations influence action selection and produce measurable differences in choice behavior. Future studies could directly test this prediction by manipulating whether emotional information is restricted to feedback or is directly relevant to action selection and fitting actor–critic models that separately estimate value‐updating and action‐selection processes. Combining this approach with feedback‐ and action‐locked neural measures would provide a direct test of whether emotional context preferentially influences critic‐like evaluation, actor‐like action selection, or both.

The present findings suggest that emotional context exerts a subtle influence on neural PE encoding rather than producing large‐scale changes in reward processing. Although emotional modulation was reliably detected at the trial level, these effects did not translate into robust differences in traditional condition‐averaged ERP measures or overt learning behavior. Furthermore, although positive and negative emotional contexts appeared to differentially influence early and later stages of feedback processing, temporal overlap between feedback‐related ERP components limits strong conclusions regarding whether these effects reflect distinct neurocognitive processes or partially overlapping neural generators (Brown and Cavanagh 2020; Krigolson 2018). Although PE‐related activity emerged most prominently within the canonical RewP time range, future studies using source localization approaches or time‐frequency analyses may help better dissociate early reward‐related processing from later evaluative feedback signals (Meyer et al. 2021). Future work may also benefit from examining dynamically fluctuating emotional states rather than discrete emotional cue categories alone, as emotional influences on reinforcement learning may emerge through ongoing fluctuations in attentional engagement, motivational state, or affective arousal over time (Chiew and Braver 2011; Xu et al. 2021).

In summary, the present study demonstrates that emotional context does not broadly alter overt learning behavior or condition‐averaged reward‐related ERP amplitudes, but instead subtly modulates the neural encoding of trialwise PEs during feedback processing. Emotional context influenced the temporal and spatial dynamics through which learning‐related neural activity evolved over time, suggesting that affective information shapes not simply whether the brain responds to reward, but how learning signals are dynamically represented and evaluated during reinforcement learning.

## Author Contributions

**Darin R. Brown:** conceptualization, investigation, funding acquisition, writing – original draft, methodology, validation, visualization, writing – review and editing, software, formal analysis, project administration, data curation, supervision, resources.

## Funding

The author has nothing to report.

## Disclosure

Methods were detailed for determining sample size, data exclusions, manipulations, and measures used in the study, in accordance with the Journal Article Reporting Standards (Appelbaum et al. 2024). All data, analysis code, and research materials are available upon request from the corresponding author. Data analysis was conducted using MATLAB, version 9.13.0 (R2022b) (MathWorks Inc 2022), and SPSS, version 29.0.2.0 (20) (IBM Corp 2023). This study's design and its analysis were not pre‐registered.

## Ethics Statement

The study was approved by the Pitzer College Institutional Review Board, Claremont Ca.

## Consent

All participants provided written informed consent.

Consent to Publish: There is no identifying information about participants included in this publication. Participants provided informed consent for publication of the results of the study.

## Conflicts of Interest

The author declares no conflicts of interest.

## Data Availability

The data that support the findings of this study are available on request from the corresponding author. The data are not publicly available due to privacy or ethical restrictions. None of the experiments were preregistered.

## References

[psyp70420-bib-0001] Appelbaum, M. , H. Cooper , R. B. Kline , E. Mayo‐Wilson , A. M. Nezu , and S. M. Rao . 2024. “Journal Article Reporting Standards for Quantitative Research in Psychology: The APA Publications and Communications Board Task Force Report.” In Methodological Issues and Strategies in Clinical Research, edited by A. E. Kazdin , 5th ed., 1031–1072. American Psychological Association. 10.1037/0000409-045. 29345484

[psyp70420-bib-0002] Bartholomeyczik, K. , M. Gusenbauer , and T. Treffers . 2022. “The Influence of Incidental Emotions on Decision‐Making Under Risk and Uncertainty: A Systematic Review and Meta‐Analysis of Experimental Evidence.” Cognition and Emotion 36, no. 6: 1054–1073. 10.1080/02699931.2022.2099349. 35838421

[psyp70420-bib-0003] Brown, D. R. , and J. F. Cavanagh . 2018. “Rewarding Images Do Not Invoke the Reward Positivity: They Inflate It.” International Journal of Psychophysiology 132: 226–235. 10.1016/j.ijpsycho.2018.02.012. PMC6686905 29505851

[psyp70420-bib-0004] Brown, D. R. , and J. F. Cavanagh . 2020. “Novel Rewards Occlude the Reward Positivity, and What to Do About It.” Biological Psychology 151: 107841. 10.1016/j.biopsycho.2020.107841. 31978500

[psyp70420-bib-0005] Brown, D. R. , T. C. Jackson , and J. F. Cavanagh . 2022. “The Reward Positivity Is Sensitive to Affective Liking.” Cognitive, Affective, & Behavioral Neuroscience 22, no. 2: 258–267. 10.3758/s13415-021-00950-5. PMC9578330 34599487

[psyp70420-bib-0006] Brown, D. R. , S. P. Richardson , and J. F. Cavanagh . 2020. “An EEG Marker of Reward Processing Is Diminished in Parkinson's Disease.” Brain Research 1727: 146541. 10.1016/j.brainres.2019.146541. 31704082

[psyp70420-bib-0007] Byrne, K. A. , Y. Liu , M. Shreeves , and S. G. Six . 2023. “Working Through Negativity : The Influence of Affective States on.” Personality and Individual Differences 201: 111949. 10.1016/j.paid.2022.111949.

[psyp70420-bib-0008] Cavanagh, J. F. , and C. B. Holroyd . 2025. “The Reward Positivity Signals a Goal Prediction Error.” Trends in Cognitive Sciences 30, no. 8: 700–711. 10.1016/j.tics.2025.11.005. PMC12768444 41483977

[psyp70420-bib-0009] Cavanagh, J. F. , S. Olguin , J. A. Talledo , et al. 2022. “Amphetamine Alters an EEG Marker of Reward Processing in Humans and Mice.” Psychopharmacology 239, no. 3: 923–933. 10.1007/s00213-022-06082-z.Amphetamine. PMC8891070 35132440

[psyp70420-bib-0010] Chase, H. W. , R. Swainson , L. Durham , L. Benham , and R. Cools . 2010. “Feedback‐Related Negativity Codes Prediction Error but Not Behavioral Adjustment During Probabilistic Reversal Learning.” Journal of Cognitive Neuroscience 23, no. 4: 936–946. 10.1162/jocn.2010.21456. 20146610

[psyp70420-bib-0011] Chiew, K. S. , and T. S. Braver . 2011. “Positive Affect Versus Reward : Emotional and Motivational Influences on Cognitive Control.” Frontiers in Psychology 2: 1–10. 10.3389/fpsyg.2011.00279. PMC3196882 22022318

[psyp70420-bib-0012] Cockburn, J. , and C. B. Holroyd . 2018. “Feedback Information and the Reward Positivity.” International Journal of Psychophysiology 132, no. November 2017: 243–251. 10.1016/j.ijpsycho.2017.11.017. 29208491

[psyp70420-bib-0013] Dayan, P. , and B. W. Balleine . 2002. “Reward, Motivation, and Reinforcement Learning.” Neuron 36, no. 2: 285–298. 10.1016/S0896-6273(02)00963-7. 12383782

[psyp70420-bib-0014] Delorme, A. , and S. Makeig . 2004. “EEGLAB: An Open Source Toolbox for Analysis of Single‐Trial EEG Dynamics.” Journal of Neuroscience Methods 13: 9–21. 10.1016/j.jneumeth.2003.10.009. 15102499

[psyp70420-bib-0015] Emanuel, A. , and E. Eldar . 2023. “Emotions as Computations.” Neuroscience & Biobehavioral Reviews 144: 104977. 10.1016/j.neubiorev.2022.104977.Emotions. PMC9805532 36435390

[psyp70420-bib-0016] Engelmann, J. B. , F. Meyer , E. Fehr , and C. C. Ruff . 2015. “Anticipatory Anxiety Disrupts Neural Valuation During Risky Choice.” Journal of Neuroscience 35, no. 7: 3085–3099. 10.1523/JNEUROSCI.2880-14.2015. PMC6605597 25698745

[psyp70420-bib-0017] Faul, F. , E. Erdfelder , A. G. Lang , and A. Buchner . 2007. “G* Power 3: A Flexible Statistical Power Analysis Program for the Social, Behavioral, and Biomedical Sciences.” Behavior Research Methods 39, no. 2: 175–191. 10.3758/BF03193146. 17695343

[psyp70420-bib-0018] Glazer, J. E. , N. J. Kelley , N. Pornpattananangkul , V. A. Mittal , and R. Nusslock . 2018. “Beyond the FRN : Broadening the Time‐Course of EEG and ERP Components Implicated in Reward Processing.” International Journal of Psychophysiology 132: 184–202. 10.1016/j.ijpsycho.2018.02.002. 29454641

[psyp70420-bib-0019] Glimcher, P. W. 2011. “Understanding Dopamine and Reinforcement Learning : The Dopamine Reward Prediction Error Hypothesis.” Proceedings of the National Academy of Sciences 108, no. supplement_3: 15647–15654. 10.1073/pnas.1014269108. PMC3176615 21389268

[psyp70420-bib-0020] Gruber, A. J. , and R. J. McDonald . 2012. “Context, Emotion, and the Strategic Pursuit of Goals: Interactions Among Multiple Brain Systems Controlling Motivated Behavior.” Frontiers in Behavioral Neuroscience 6: 50. 10.3389/fnbeh.2012.00050. PMC3411069 22876225

[psyp70420-bib-0021] Harlé, K. M. , P. Shenoy , and M. P. Paulus . 2013. “The Influence of Emotions on Cognitive Control : Feelings and Beliefs — Where Do They Meet ?” Frontiers in Human Neuroscience 7, no. 508: 1–16. 10.3389/fnhum.2013.00508. PMC3776943 24065901

[psyp70420-bib-0022] Harmon‐jones, E. , P. A. Gable , and T. F. Price . 2012. “The Influence of Affective States Varying in Motivational Intensity on Cognitive Scope.” Frontiers in Integrative Neuroscience 6, no. 73: 1–5. 10.3389/fnint.2012.00073. PMC3437552 22973207

[psyp70420-bib-0023] Heffner, J. , R. Frömer , O. Feldmanhall , and M. R. Nassar . 2025. “Separable Neural Signals for Reward and Emotion Prediction Errors.” Nature Communications 16, no. 1: 1–11. 10.1038/s41467-025-63135-5. PMC12373756 40846846

[psyp70420-bib-0024] Holroyd, C. B. , T. E. Baker , K. A. Kerns , and U. Müller . 2008. “Electrophysiological Evidence of Atypical Motivation and Reward Processing in Children With Attention‐Deficit Hyperactivity Disorder.” Neuropsychologia 46, no. 8: 2234–2242. 10.1016/j.neuropsychologia.2008.02.011. 18367216

[psyp70420-bib-0025] Holroyd, C. B. , and M. G. H. Coles . 2002. “The Neural Basis of Human Error Processing: Reinforcement Learning, Dopamine, and the Error‐Related Negativity.” Psychological Review 109: 679–709. 10.1037//0033-295X.109.4.679. 12374324

[psyp70420-bib-0026] Houk, J. C. , J. L. Adams , and A. G. Barto . 1995. “A Model of How the Basal Ganglia Generate and Use Neural Signals That Predict Reinforcement.” In Models of Information Processing in the Basal Ganglia, edited by J. C. Houk , J. L. Davis , and D. G. Beiser , 249–270. MIT Press.

[psyp70420-bib-0027] Hoy, C. W. , S. C. Steiner , and R. T. Knight . 2021. “Single‐Trial Modeling Separates Multiple Overlapping Prediction Errors During Reward Processing in Human EEG.” Communications Biology 4, no. 1: 1–17. 10.1038/s42003-021-02426-1. PMC8302587 34302057

[psyp70420-bib-0028] IBM Corp . 2023. IBM SPSS Statistics for Windows, Version 29.0.2.0. IBM Corp.

[psyp70420-bib-0029] Isbell, L. M. , E. C. Lair , and D. R. Rovenpor . 2013. “Affect‐As‐Information About Processing Styles : A Cognitive Malleability Approach.” Social and Personality Psychology Compass 7, no. 2: 93–114. 10.1111/spc3.12010.

[psyp70420-bib-0030] Jackson, T. C. J. , and J. F. Cavanagh . 2023. “Reduced Positive Affect Alters Reward Learning via Reduced Information Encoding in the Reward Positivity.” Psychophysiology 60, no. 8: 1–16. 10.1111/psyp.14276. 36807324

[psyp70420-bib-0031] John, Y. J. , D. Bullock , B. Zikopoulos , and H. Barbas . 2013. “Anatomy and Computational Modeling of Networks Underlying Cognitive‐Emotional Interaction.” Frontiers in Human Neuroscience 7, no. 101: 1–26. 10.3389/fnhum.2013.00101. PMC3613599 23565082

[psyp70420-bib-0032] Katahira, K. , Y.‐T. Matsuda , T. Fujimura , et al. 2015. “Neural Basis of Decision Making Guided by Emotional Outcomes.” Journal of Neurophysiology 113, no. 9: 3056–3068. 10.1152/jn.00564.2014. PMC4455562 25695644

[psyp70420-bib-0033] Kool, W. , S. J. Getz , and M. M. Botvinick . 2013. “Neural Representation of Reward Probability: Evidence From the Illusion of Control.” Journal of Cognitive Neuroscience 25, no. 6: 852–861. 10.1162/jocn. 23410034

[psyp70420-bib-0034] Krigolson, O. E. 2018. “Event‐Related Brain Potentials and the Study of Reward Processing : Methodological Considerations ☆.” International Journal of Psychophysiology 132: 175–183. 10.1016/j.ijpsycho.2017.11.007. 29154804

[psyp70420-bib-0035] Lang, P. J. , M. M. Bradley , and B. N. Cuthbert . 2005. International Affective Picture System (IAPS): Affective Ratings of Pictures and Instruction Manual (Technical Report No. A‐6). University of Florida, Center for the Study of Emotion & Attention.

[psyp70420-bib-0036] Lefebvre, G. , M. Lebreton , F. Meyniel , S. Bourgeois‐Gironde , and S. Palminteri . 2017. “Behavioural and Neural Characterization of Optimistic Reinforcement Learning.” Nature Human Behaviour 1, no. 4: 1–9. 10.1038/s41562-017-0067.

[psyp70420-bib-0037] Lench, H. C. , K. E. Darbor , and L. A. Berg . 2013. “Functional Perspectives on Emotion, Behavior, and Cognition.” Behavioral Science 3, no. 4: 536–540. 10.3390/bs3040536. PMC4217601 25379254

[psyp70420-bib-0038] Liu, F. , Y. Jiang , and B. Du . 2025. “Neuropsychologia Reward Prediction‐Error Promotes the Neural Encoding of Episodic Learning.” Neuropsychologia 211: 109120. 10.1016/j.neuropsychologia.2025.109120. 40073967

[psyp70420-bib-0039] Lohse, K. R. , M. W. Miller , M. Daou , W. Valerius , and M. Jones . 2020. “Dissociating the Contributions of Reward‐Prediction Errors to Trial‐Level Adaptation and Long‐Term Learning.” Biological Psychology 149: 107775. 10.1016/j.biopsycho.2019.107775. 31563586

[psyp70420-bib-0072] Makeig, S. , A. J. Bell , T.‐P. Jung , and T. J. Sejnowski . 1996. “Independent Component Analysis of Electroencephalographic Data.” Advances in Neural Information Processing Systems 8: 145–151. 10.1109/ICOSP.2002.1180091.

[psyp70420-bib-0040] MathWorks Inc . 2022. MATLAB Version: 9.13.0 (R2022b) Natick, Massachusetts: The MathWorks Inc. https://www.mathworks.com.

[psyp70420-bib-0041] Meyer, G. M. , J. Marco‐pallarés , and P. Boulinguez . 2021. “Electrophysiological Underpinnings of Reward Processing : Are We Exploiting the Full Potential of EEG?” NeuroImage 242: 118478. 10.1016/j.neuroimage.2021.118478. 34403744

[psyp70420-bib-0042] Nasser, H. M. , D. J. Calu , G. Schoenbaum , and M. J. Sharpe . 2017. “The Dopamine Prediction Error : Contributions to Associative Models of Reward Learning.” Frontiers in Psychology 8, no. 244: 1–17. 10.3389/fpsyg.2017.00244. PMC5319959 28275359

[psyp70420-bib-0043] Niv, Y. , J. A. Edlund , P. Dayan , and J. P. O. Doherty . 2012. “Neural Prediction Errors Reveal a Risk‐Sensitive Reinforcement‐Learning Process in the Human Brain.” Journal of Neuroscience 32, no. 2: 551–562. 10.1523/JNEUROSCI.5498-10.2012. PMC6621075 22238090

[psyp70420-bib-0044] Nolan, H. , R. Whelan , and R. B. Reilly . 2010. “FASTER: Fully Automated Statistical Thresholding for EEG Artifact Rejection.” Journal of Neuroscience Methods 192, no. 1: 152–162. 10.1016/j.jneumeth.2010.07.015. 20654646

[psyp70420-bib-0045] O'doherty, J. , P. Dayan , J. Schultz , R. Deichmann , K. Friston , and R. J. Dolan . 2004. “Dissociable Roles of Ventral and Dorsal Striatum in Instrumental.” Science 304, no. 5669: 452–454. 10.1126/science.1094285. 15087550

[psyp70420-bib-0046] Pagan, M. , V. D. Tang , M. C. Aoi , et al. 2025. “Individual Variability of Neural Computations Underlying Flexible Decisions.” Nature 639, no. 8054: 421–429. 10.1038/s41586-024-08433-6. PMC11903320 39608399

[psyp70420-bib-0047] Phelps, E. A. , K. M. Lempert , and P. Sokol‐hessner . 2014. “Emotion and Decision Making : Multiple Modulatory Neural Circuits.” Annual Review of Neuroscience 37, no. 1: 263–287. 10.1146/annurev-neuro-071013-014119. 24905597

[psyp70420-bib-0048] Pike, A. C. , and O. J. Robinson . 2022. “Reinforcement Learning in Patients With Mood and Anxiety Disorders vs Control Individuals A Systematic Review and Meta‐Analysis.” JAMA Psychiatry 79, no. 4: 313–322. 10.1001/jamapsychiatry.2022.0051. PMC8892374 35234834

[psyp70420-bib-0049] Polich, J. 2017. “Updating P300: An Integrative Theory of P3a and P3b.” Clinical Neurophysiology 118, no. 10: 2128–2148. 10.1016/j.clinph.2007.04.019.Updating. PMC2715154 17573239

[psyp70420-bib-0050] Proudfit, G. H. 2015. “The Reward Positivity: From Basic Research on Reward to a Biomarker for Depression.” Psychophysiology 52, no. 4: 449–459. 10.1111/psyp.12370. 25327938

[psyp70420-bib-0051] Puccetti, N. A. , W. J. Villano , J. P. Fadok , and A. S. Heller . 2022. “Temporal Dynamics of Affect in the Brain: Evidence From Human Imaging and Animal Models.” Neuroscience & Biobehavioral Reviews 133: 104491. 10.1016/j.neubiorev.2021.12.014. PMC8792368 34902442

[psyp70420-bib-0052] Pulcu, E. , W. Lin , S. Han , and M. Browning . 2024. “Depression Is Associated With Reduced Outcome Sensitivity in a Dual Valence, Magnitude Learning Task.” Psychological Medicine 54, no. 3: 631–636. 10.1017/S0033291723002520. PMC11443165 37706290

[psyp70420-bib-0053] Roese, N. J. , and J. M. Olson . 2007. “Better, Stronger, Faster Self‐Serving Judgment, Affect Regulation, and the Optimal Vigilance Hypothesis.” Perspectives on Psychological Science 2, no. 2: 124–141. 10.1111/j.1745-6916.2007.00033.x. PMC2429993 18552989

[psyp70420-bib-0054] Rubin, A. , L. Sheintuch , N. Brande‐eilat , et al. 2019. “Revealing Neural Correlates of Behavior Without Behavioral Measurements.” Nature Communications 10, no. 1: 1–14. 10.1038/s41467-019-12724-2. PMC6802184 31628322

[psyp70420-bib-0055] Sajda, P. , M. G. Philiastides , and L. C. Parra . 2009. “Single‐Trial Analysis of Neuroimaging Data: Inferring Neural Networks Underlying Perceptual Decision‐Making in the Human Brain.” IEEE Reviews in Biomedical Engineering 2: 97–109. 10.1109/RBME.2009.2034535. PMC3477547 22275042

[psyp70420-bib-0056] Sambrook, T. D. , and J. Goslin . 2015. “A Neural Reward Prediction Error Revealed by a Meta‐Analysis of ERPs Using Great Grand Averages.” Psychological Bulletin 141, no. 1: 213–235. 10.1037/bul0000006. 25495239

[psyp70420-bib-0057] Sander, D. , and L. Nummenmaa . 2021. “Reward and Emotion: An Affective Neuroscience Approach.” Current Opinion in Behavioral Sciences 39: 161–167. 10.1016/j.cobeha.2021.03.016.

[psyp70420-bib-0058] Schmidt, L. , E. K. Braun , T. D. Wager , and D. Shohamy . 2014. “Mind Matters: Placebo Enhances Reward Learning in Parkinson 's Disease.” Nature Neuroscience 17, no. 12: 1793–1797. 10.1038/nn.3842. PMC4503317 25326691

[psyp70420-bib-0059] Shalev, T. , and B. Sheva . 2025. “Modulation of Perception by Emotion : Altered Sensitivity and Perceived Magnitude of Negatively Valenced Stimuli.” Attention, Perception, & Psychophysics 87, no. 6: 1964–1973. 10.3758/s13414-025-03110-w. PMC12331848 40640489

[psyp70420-bib-0060] Stange, J. P. , A. Macnamara , A. E. Kennedy , et al. 2020. “Neuropsychologia Brain‐Behavioral Adaptability Predicts Response to Cognitive Behavioral Therapy for Emotional Disorders : A Person‐Centered Event‐Related Potential.” Neuropsychologia 145: 106408. 10.1016/j.neuropsychologia.2017.06.027. PMC5742079 28648570

[psyp70420-bib-0061] Sutton, R. S. , and A. G. Barto . 1981. “Toward a Modern Theory of Adaptive Networks : Expectation and Prediction.” Psychological Review 88, no. 2: 135–170. 10.1037/0033-295X.88.2.135. 7291377

[psyp70420-bib-0062] Teoh, Y. Y. , S. Reisman , J. Heffner , and O. FeldmanHall . 2026. “Affective Valence as a Computational Signal for Learning Value.” Communications Psychology 4: 101. 10.1038/s44271-026-00458-z. PMC13333503 42056466

[psyp70420-bib-0063] Tunison, E. , R. Sylvain , J. Sterr , V. Hiley , and J. M. Carlson . 2019. “No Money, no Problem: Enhanced Reward Positivity in the Absence of Monetary Reward.” Frontiers in Human Neuroscience 13: 1–5. 10.3389/fnhum.2019.00041. PMC6379288 30809138

[psyp70420-bib-0064] Vogel, B. O. , C. Shen , and A. H. Neuhaus . 2015. “Emotional Context Facilitates Cortical Prediction Error Responses.” Human Brain Mapping 36, no. 9: 3641–3652. 10.1002/hbm.22868. PMC6869547 26047176

[psyp70420-bib-0065] Vollberg, M. C. , and D. Sander . 2024. “Hidden Reward : Affect and Its Prediction Errors as Windows Into Subjective Value.” Current Directions in Psychological Science 33, no. 2: 93–99. 10.1177/09637214231217678. PMC10981566 38562909

[psyp70420-bib-0066] Watanabe, N. , M. Sakagami , and M. Haruno . 2013. “Reward Prediction Error Signal Enhanced by Striatum‐Amygdala Interaction Explains the Acceleration of Probabilistic Reward Learning by Emotion.” Journal of Neuroscience 33, no. 10: 4487–4493. 10.1523/JNEUROSCI.3400-12.2013. PMC6704947 23467364

[psyp70420-bib-0067] Weber, C. , and C. Bellebaum . 2024. “Prediction ‐ Error ‐ Dependent Processing of Immediate and Delayed Positive Feedback.” Scientific Reports 14, no. 1: 1–15. 10.1038/s41598-024-60328-8. PMC11055855 38678065

[psyp70420-bib-0068] Xu, S. , Y. Sun , M. Huang , et al. 2021. “Emotional State and Feedback‐Related Negativity Induced by Positive, Negative, and Combined Reinforcement.” Frontiers in Psychology 12: 1–13. 10.3389/fpsyg.2021.647263. PMC8141566 34040560

[psyp70420-bib-0069] Xue, S. , J. Cui , K. Wang , S. Zhang , J. Qiu , and Y. Luo . 2013. “Positive Emotion Modulates Cognitive Control : An Event‐Related Potentials Study.” Scandinavian Journal of Psychology 54, no. 2: 82–88. 10.1111/sjop.12031. 23397988

[psyp70420-bib-0070] Yiend, J. 2010. “The Effects of Emotion on Attention: A Review of Attentional Processing of Emotional Information.” Cognition and Emotion 24, no. 1: 3–47. 10.1080/02699930903205698.

[psyp70420-bib-0071] Zhang, J. , B. Paul‐christian , A. Kiesel , and D. Dignath . 2023. “How Emotional Stimuli Modulate Cognitive Control : A Meta‐Analytic Review of Studies With Con Fl Ict Tasks.” Psychological Bulletin 149, no. 2: 25–66.

